# Comparison of the McGrath MAC video laryngoscope and the Pentax Airwayscope during chest compression: a manikin study

**DOI:** 10.1186/2052-0492-2-18

**Published:** 2014-03-06

**Authors:** Atsushi Kotera, Hiroki Irie, Shinsuke Iwashita, Junichi Taniguchi, Shunji Kasaoka, Yoshihiro Kinoshita

**Affiliations:** Department of Emergency and General Medicine, Kumamoto University Hospital, 1-1-1 Honjo, Chuo-ku, Kumamoto City, Kumamoto Prefecture, 860-8556 Japan; Department of Intensive Care Unit, Kumamoto University Hospital, 1-1-1 Honjo, Chuo-ku, Kumamoto City, Kumamoto Prefecture, 860-8556 Japan

**Keywords:** McGrath MAC, Pentax AirwayScope, Chest compression

## Abstract

We tested the utility of the McGrath MAC^®^ (McG) video laryngoscope during chest compression compared with the Pentax Airwayscope^®^ (AWS). We recruited 59 participants into the simulation study. The difference in the time to intubation (TTI [sec]) between without and with chest compression was significant for the AWS attempts (median 13, range 6–28 vs. median 15, range 6–72, *p* = 0.0247) but not significant for the McG attempts (median 16, range 6–75 vs. median 16, range 6–71); however, the difference of the TTIs is not serious clinically. The utility of the two devices during chest compressions is almost similar although their characteristics are different.

## Introduction

The Pentax Airwayscope^®^ (AWS; Pentax, Tokyo, Japan) can improve the view of the glottis during chest compression [1, 2]. However, the AWS occasionally contacts the arm of the chest compressor because of its large body [3]. We constructed a hypothesis that a compact device could facilitate the intubation attempt during chest compression. The McGrath MAC^®^ video laryngoscope (McG; Covidien, Tokyo, Japan) is a new developed compact device. Here, we compared the utility of the two devices.

## Findings

We recruited the 59 participants (51 medical university students and eight medical vocational college students) into the simulation study. We used the Airman (Laerdal, Sentrum and Stavanger, Norway) as the manikin model. The participants performed intubation attempts in the following order: AWS without chest compression, AWS with chest compression, McG without chest compression, and McG with chest compression. Chest compression was performed by an Advanced Cardiac Life Support provider. We used an endotracheal tube (ETT; Portex, St. Paul, MN, USA) with an internal diameter of 7.0 mm.

We measured the time to tracheal intubation (TTI) of each intubation attempt. For the McG attempts, the TTI was defined as the duration from grasping the device to removing the metal stylet from the ETT. For the AWS attempts, the TTI was defined as the duration from grasping the device to removing the blade from the manikin's mouth. We defined ‘failed tracheal intubation’ as either esophageal intubation or exceeding the time limit of 90 s for the attempt. We used the Mann-Whitney *U* test to test for differences in the TTIs, and we considered *p* values <0.05 significant.

**Figure 1:**
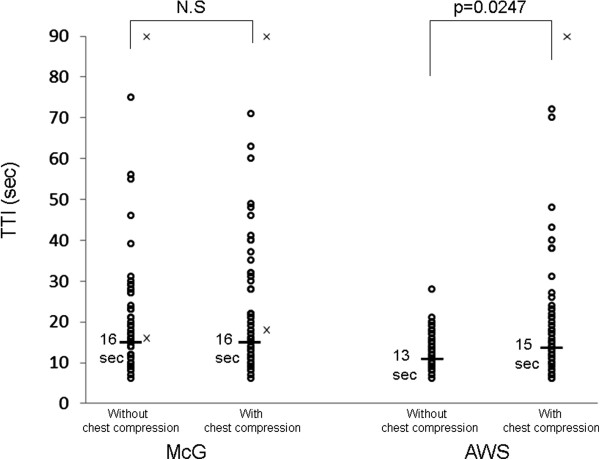
**Scatter graph of TTI in all of participants using each device without and with chest compression.** The thick bar indicates the median TTI. Open circles indicate successful tracheal intubations, and x indicates a failed tracheal intubation.

The TTIs for each device are shown (Figure 1). The difference in the TTI (sec) between without and with chest compression was significant for the AWS attempts (median 13, range 6–28 vs. median 15, range 6–72, *p* = 0.0247) but not significant for the McG attempts (median 16, range 6–75 vs. median 16, range 6–71). Two participants failed the intubation attempt in the McG attempts in each situation, and one participant in the AWS attempts in a situation with chest compression (Figure 1).

**Figure 2:**
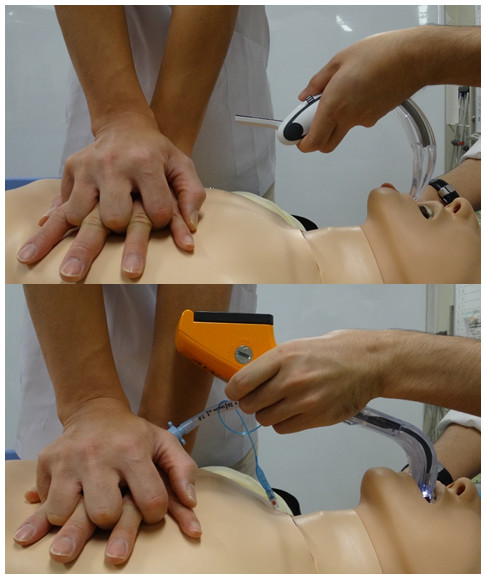
**McG does not contact arm of chest compressor at insertion, but AWS does.**

In general, in attempts with video laryngoscopes, the relative positions of the glottis and ETT in a monitor are stable during chest compression [4, 5]. However, the characteristics of each video laryngoscope are different and each device has its own advantages and disadvantages.The distance from the device to the arm of the chest compressor differed between the two devices (Figure 2). In the present study, 42 of the 59 participants inserted the AWS obliquely into the manikin's mouth in order to not contact the arm of the chest compressor. The oblique insertion of the AWS took more time. Conversely, the operator could insert the McG in a straight manner because of its compact body. The AWS's large body may contribute to the prolonged TTIs during chest compression. However, the difference of the TTIs is 2 s, and that is not serious in a clinical situation. Additionally, there was no significant difference in the TTIs with chest compression in the attempts using the two devices. Considering our data, a compact device may not always facilitate the intubation attempt during chest compression.

The present study has several limitations. First, the manikin model cannot reproduce the precise intubation conditions of real patients. Second, chest compressions on a manikin model cannot duplicate CPR on real patients. Third, the participants are students who do not have enough skills needed for tracheal intubation. Fourth, the present study is not a randomized crossover study, and improvement in the participants' skills as each attempt progressed may have occurred.
